# pH-Sensitive Mixed Micelles Assembled from PDEAEMA-PPEGMA and PCL-PPEGMA for Doxorubicin Delivery: Experimental and DPD Simulations Study

**DOI:** 10.3390/pharmaceutics12020170

**Published:** 2020-02-18

**Authors:** Chufen Yang, Wenyao Liu, Jiayu Xiao, Cong Yuan, Yaoxi Chen, Jianwei Guo, Hangbo Yue, Dongyu Zhu, Wenjing Lin, Shengqiu Tang, Xiaoying Dong

**Affiliations:** 1 School of Chemical Engineering and Light Industry, Guangdong University of Technology, Guangzhou 510006, China; cfyang@gdut.edu.cn (C.Y.); wyliu11@126.com (W.L.); 13128263809@163.com (J.X.); yuancong168524@163.com (C.Y.); 13719339548@163.com (Y.C.); guojw@gdut.edu.cn (J.G.); hangbo.yue@gdut.edu.cn (H.Y.); zdy16@gdut.edu.cn (D.Z.); 2 Guangdong Provincial Key Lab of Green Chemical Product Technology, School of Chemistry and Chemical Engineering, South China University of Technology, Guangzhou 510640, China; 3 College of Yingdong Agricultural Science and Engineering, Shaoguan University, Shaoguan 512005, China; willertang@163.com; 4 Guangdong Province Key Laboratory of Animal Nutritional Regulation, South China Agricultural University, Guangzhou 510642, China

**Keywords:** drug delivery system, pH-responsive, mixed micelles, dissipative particle dynamics simulation, well-controlled release

## Abstract

To decrease critical micelle concentration (CMC), improve stability, and keep high drug-loading capacity, three pH-sensitive mixed micelles applied for anticancer drug controlled delivery were prepared by the mixture of polymers poly (*N,N*-diethylaminoethyl methacrylate)-*b*-poly(poly(ethylene glycol) methyl ether methacrylate) (PDEAEMA-PPEGMA) and polycaprolactone-*b*-poly (poly(ethylene glycol) methyl ether methacrylate) (PCL-PPEGMA), which were synthesized and confirmed by ^1^H NMR and gel permeation chromatographic (GPC). The critical micelle concentration (CMC) values of the prepared mixed micelles were low, and the micellar sizes and zeta potentials of the blank mixed micelles demonstrated good pH-responsive behavior. Combined experimental techniques with dissipative particle dynamics (DPD) simulation, the particle sizes, zeta potentials, drug loading content (LC), encapsulation efficiency (EE), aggregation morphologies, and doxorubicin (DOX) distribution of the mixed micelles were investigated, and the high DOX-loading capacity of the mixed micelles was found. Both in vitro DOX release profiles and DPD simulations of the DOX dynamics release process exhibited less leakage and good stability in neutral conditions and accelerated drug release behavior with a little initial burst in slightly acidic conditions. Cytotoxicity tests showed that the polymer PDEAEMA-PPEGMA and the blank mixed micelles had good biocompatibility, and DOX-loaded mixed micelles revealed certain cytotoxicity. These results suggest that the drug-loaded mixed micelles that consisted of the two polymers PDEAEMA-PPEGMA and PCL-PPEGMA can be new types of pH-responsive well-controlled release anticancer drug delivery mixed micelles.

## 1. Introduction

In recent years, nanoscopic polymeric micelles self-assembled by amphiphilic polymers have emerged as prospective nanocarriers for drug delivery, which show unique advantages in enhancing drug bioavailability, improving therapeutic efficacy, and lowering side effects [1,2,3,4,5]. Generally, these typical polymeric micelles have a core–shell structure because a hydrophobic inner core can encapsulate hydrophobic drugs, and the outer hydrophilic shell keeps the system stable in the long-time circulation and decreases the toxicity of drugs in the human body [6,7,8,9]. Furthermore, as a promising and effective drug delivery vehicle, nanoscopic polymeric micelles should also have the ability to remain stable in normal tissues and release the majority of the loaded drug into the diseased tissues in a well-controlled manner. Consequently, more and more attention has been paid to stimuli-responsive nanocarriers in the last few years [10,11,12,13]. Thereinto, it is confirmed that the pH-sensitive amphiphilic polymers micelles have smart abilities in drug-controlled release, which can change their conformation upon pH variation easily, thus offering a targeted and controlled method for the delivery of wrapped drugs [14,15].

The different pH-sensitive polymeric micelles can be designed for release to different organs, tissues, and cells in a well-controlled manner. As for delivering intravenous anticancer drugs, it tends to require that the pH-triggered amphiphilic copolymers micelles remain stable in normal tissues (pH 7.4), while releasing the anticancer drugs quickly in tumor tissue (pH 5.0–6.5) [16,17,18,19,20]. Although great achievements have been made, there are still several significant parameters that need to be further improved and optimized, such as the micellar targetability, stability, longevity, drug loading capacity, particle sizes, and size distribution. Recently, mixing two or more dissimilar species of block polymers has been found to be a straightforward and potent strategy for improving some of the performance of the pH-sensitive drug-loaded polymeric micelles [21,22,23]. Bae et al. prepared a series of drug-loaded mixed micelles polyhistidine-polyethylene glycol/polylactic acid-polyethylene glycol [polyHis-PEG/PLA-PEG], and they found that these mixed micelles exhibited good pH-response release ability and prolonged stability and longevity [24]. In Leroux’s work, the mixed block polymeric micelles composed of poly(ethylene glycol)-*b*-poly(D-lactide) (PEG-PDLA) and poly(ethylene glycol)-*b*-poly(L-lactide) (PEG-PLLA) showed better performance [good drug loading content (LC), great encapsulation efficiency (EE), and well-controlled release rates] than either of the polymers alone [25].

In our previous work, we developed two diblock polymers—poly (ethylene glycol) methyl ether-*b*-polycaprolactone (MPEG-PCL) and poly (ethylene glycol) methyl ether-*b*-poly (*N*,*N*-iethylamino ethyl methacrylate) (MPEG-PDEAEMA)—and used their mixed micelles to load doxorubicin (DOX) and found high DOX loading efficiency and good pH sensitivity [26]. However, the critical micelle concentration (CMC) values of these mixed micelles were found to be considerably high, leading to the relatively lower stability, more leakage in neutral conditions, and a deficient desired drug release performance.

**Scheme 1:**
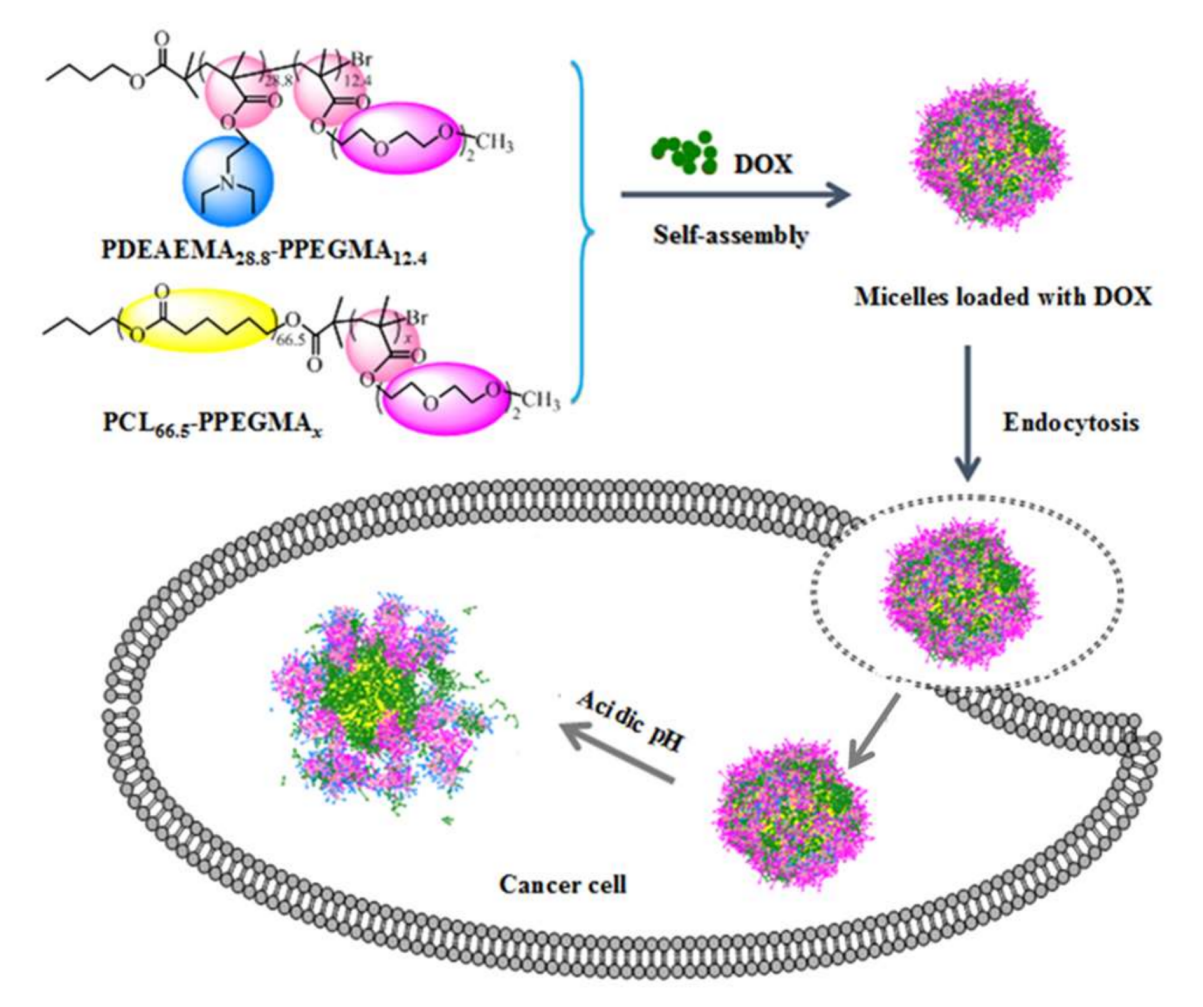
Scheme of DOX-loaded mixed micelles formation and pH-sensitive release of DOX.

Based on that, in this study, our efforts focused on decreasing the CMC values of the mixed micelles while maintaining their high LC and EE performance, as well as obtaining well-controlled drug release performance. To achieve this goal, the novel self-assembled mixed micelles from two diblock polymers, poly (*N,N*-diethylaminoethyl methacrylate)-*b*-poly(poly(ethylene glycol) methyl ether methacrylate) (PDEAEMA-PPEGMA) and polycaprolactone-*b*-poly (poly(ethylene glycol) methyl ether methacrylate) (PCL-PPEGMA), were employed as the new drug delivery system. In this system, the hydrophobic PCL block was used as a hydrophobic block for its excellent biocompatibility in biomedical applications, which formed the micelles core and encapsulated the hydrophobic drug [27,28]. Instead of the linear hydrophilic MPEG in the previous work, the hydrophilic brush PEGMA with short side chains, whose dense brush structure might decrease the CMC values of micelles, was selected to distribute on the outer surface of the self-assembled micelles and maintain the stability of micelles during biological circulation [29,30,31,32]. The DEAEMA block, whose p*K*_b_ was about 6.9—making it soluble in relatively weak acidic conditions but insoluble in normal physiological conditions—was used as the pH-responsive block [33,34,35]. As among the most effective anticancer drugs, DOX was utilized as the model drug and loaded into the mixed micelles. Scheme 1 presented the schematic of the encapsulation and pH-sensitive DOX release from the mixed micelles. The mixture of polymers PDEAEMA-PPEGMA and PCL-PPEGMA, together with DOX, were self-assembled to form high biophysically stable DOX-loaded mixed micelles in neutral aqueous solution, which entered the cancer cells through endocytosis, and then released DOX in a well-controlled rate in the mildly acidic endosome/lysosome environment. To investigate its potential as the pH-responsive anticancer delivering carriers, the micellar sizes, and the zeta potential’s dependence on pH values, the LC, EE, and other physicochemical performances as well as in vitro DOX release and cytotoxicity of the mixed micelles were investigated in detail.

As the delivery of the drug-loaded polymeric micelles involves a complex polydisperse multiphase system focused on nanoscopic scales, a lot of mesoscopic information about the drug-loaded micelles is difficult to obtain using only experimental techniques. In this paper, dissipative particle dynamics (DPD) simulation was employed to explore the self-assembled behavior of the DOX-loaded mixed micelles, their microstructures, and detailed DOX distribution, as well as the dynamics release process of DOX, hoping to enable further understanding of the structure–performance relationship for the controlled and sustained drug delivery as the auxiliary of the experimentally derived results [36,37].

## 2. Materials and Methods

### 2.1. Materials

Poly (ethylene glycol) methyl ether methacrylate) (PEGMA, *M*_n_=300 Da, 99%, Aldrich, St. Louis, MO, USA) was purified firstly by passing through a column filled with neutral alumina before use. *N,N*-diethylaminoethyl methacrylate (DEAEMA, 98%) and ε-caprolactone (ε-CL, 99%) were purchased from Mackin Co. Ltd. (Shanghai, China). Triethylamine (TEA, 99%, Aldrich), tetrahydrofuran (THF, AR, Energy Chemical, Shanghai, China), dichloromethane (DCM, AR, Energy Chemical), n-butanol, and toluene were mixed with suitable calcium hydride (CaH_2_) and stirred for 24 h; then, they were distilled under atmospheric pressure and sealed preservation. Hydrochloric acid in doxorubicin hydrochloride (DOX·HCl) (Wuhan Yuancheng Gongchuang Technology Co. Ltd., Wuhan, China) was removed by TEA before use. 2-Bromoisobutyryl bromide (BIBB, 98%), pentamethyl diethylenetriamine (PMDETA, 99%), pyrene, and stannous octoate (Sn(Oct)_2_) were obtained from Mackin Co. Ltd.(Shanghai, China) and utilized as received. Dimethyl sulfoxide (DMSO), cupric bromide (CuBr_2_), and 3-(4,5-Dimethylthiazol-2-yl)-2,5-diphenyltetrazoxium bromide (MTT) were bought from Sigma Chemical Co. (St. Louis, MO, USA). HepG2 cells were bought from the American Type Culture Collection (ATCC, Manassas, VA, USA) and cultured according to the instruction manual. All other reagents were used without any further treatment.

### 2.2. Synthesis of PDEAEMA-PPEGMA

Using n-butanol 2-bromoisobutyrate as an initiator, the PDEAEMA-PPEGMA with pH-responsive performance was synthesized through the activator regenerated by electron transfer atom transfer radical polymerization (ARGET ATRP) method. The initiator was synthesized as follows: n-butanol (4.575 mL, 0.05 mol) and THF (60 mL) were added to a 250 mL flame-dried Schlenk flask and mixed uniformity. Then, we placed the whole system in an ice bath and purged it with argon thrice. TEA (6.955 mL, 0.05 mol) and 2-bromoisobutyryl bromide (6.183 mL, 0.05 mol) were successively injected dropwise into the stirred mixture followed by 5 h at 0 °C and a continued 24 h at 25 °C reaction. After THF was evaporated by rotary evaporation, the rude product was dissolved in DCM. Then, the DCM layer was serially washed with diluted HCl, NaHCO_3_, and distilled water. The organic phase was dried with MgSO_4_ for 12 h, and then all the DCM was removed by rotary evaporation to obtain the small molecule initiator n-butanol 2-bromoisobutyrate.

The synthetic procedure of PDEAEMA-PPEGMA was as follows: n-butanol 2-bromoisobutyrate (0.233 g, 1.0 mmol) and CuBr_2_ (26.34 mg) were charged into another 150 mL Schlenk flask equipped with a magnetic stirring bar, which was pumped with argon three times to remove oxygen. DEAEMA (6.63 g, 35 mmol) and PMDETA (0.208 mL, 1.0 mmol) were orderly dissolved in 30 mL of THF and injected in the flask. Then, Sn(Oct)_2_ (0.39 g, 1.0 mmol) previously dissolved in 2 mL of THF was added dropwise in the flask. After stirring the mixture for 15 min, the polymerization was performed in the 60 °C thermostatic oil bath for 6 h. Subsequently, PEGMA (4.59 g, 15 mmol) dissolved in 5 mL of THF was injected in the flask, and the reaction was continued for 24 h. After that, the mixture was cooled to around room temperature and then diluted in THF (50 mL) to purify by passing through a neutral alumina column, followed by rotary evaporation to remove the THF. The remained crude product was precipitated in cold n-hexane, filtered, and then dried at 45 °C for 48 h under vacuum. Finally, the product of diblock polymer PDEAEMA-PPEGMA was obtained.

### 2.3. Synthesis of PCL-PPEGMA

The PCL-PPEGMA was synthesized via the ring opening polymerization (ROP) of ε-CL followed by ARGET ATRP of PEGMA. (1) Using n-butanol as the initiator, PCL-OH was prepared through the ROP method. N-butanol (0.149 g, 2 mmol) and ε-CL (11.414 g, 0.1 mol) were placed in a 150 mL dried round-bottom flask that was evacuated and flushed with argon three times. Sn(Oct)_2_ (0.1141 g, 0.1 wt% of ε-CL) dissolved in 1 mL of anhydrous toluene, injected in the flask, and then carried out in an oil bath at 130 °C for 24 h. After the reaction, the product was diluted in THF (20 mL), and then added dropwise to the cold water/methanol mixture (*v*/*v* = 1:1). The resulting product PCL-OH was obtained by suction filtration and dried at 40 °C under vacuum for 48 h. (2) Equipped with a magnetic stirring bar, PCL-OH (6.3 g, 1 mmol) and THF (30 mL) were injected into a 100 mL flame-dried Schlenk flask under argon atmosphere. After the polymer dissolved, TEA (0.46 mL, 4 mmol) and BIBB (0.42 mL, 4 mmol) were injected dropwise into the flask successively under ice bath conditions (0 °C) while being vigorously stirred for 4 h and then warmed up to 25 °C for 24 h. Afterwards, the mixture was filtered by passing through a neutral alumina column, and the THF was removed under rotary evaporation; then, it was precipitated twice by cold n-hexane to collect PCL-Br. (3) Synthesis of PCL-PPEGMA: THF (30 mL), PCL-Br (6.5 g, 1 mmol), and CuBr_2_ (0.018 g, 0.08 mmol) were added in a 100 mL dried Schlenk flask equipped with a magnetic stirring bar under argon. PEGMA (6.1 mL, 20 mmol) and PMDETA (0.167 mL, 0.8 mmol) were introduced into the flask with syringes in turn. Sn(Oct)_2_ (0.39 g, 1.0 mmol) dissolved in THF (2 mL) was added into the flask. After the reaction was carried out in an oil bath at 60 °C for 24 h, the mixture was dissolved in 30 mL of THF and flowed through an alumina column to remove CuBr_2_; then, the solution was concentrated by a rotation-evaporation process. Finally, it was precipitated into cold n-hexane dropwise and dried under vacuum at 40 °C for 48 h to obtain the product PCL-PPEGMA.

### 2.4. Characterization and Measurement

The structures of PCL-OH, PDEAEMA-PPEGMA, and PCL-PPEGMA were confirmed by a Bruker AVANCE 400-MHz NMR spectrometer, which was operated using CDCl_3_ as the solvent at 25 °C. The molecular weight (*M*_n_) and the dispersity index (*M*_w_/*M*_n_) of the PCL-OH, PDEAEMA-PPEGMA, and PCL-PPEGMA were measured by gel permeation chromatographic (GPC) on an Agilent 1200 GPC equipment using THF as the eluent. The CMC values of PDEAEMA-PPEGMA, PCL-PPEGMA, and their mixture were determined by a Japanese Hitachi F-4500 fluorescence spectrometer with an emission wavelength at 373 nm and bandwidth of 0.4 nm at room temperature. The particle sizes, size distribution (PDI), and zeta potentials of the blank mixed micelles and DOX-loaded mixed micelles were measured by dynamic light scattering (DLS) instrument. Morphologies of the samples were obtained from transmission electron microscopy (TEM, FEI, Hillsboro, OR, USA) operated at 120 kV.

### 2.5. Determination of CMC Values

Through the usage of pyrene as the fluorescence probe, the CMC values of mixed micelles were measured by the fluorescence probe technique. The determined procedure was simplified as follows: First, a series of solutions formed by the polymers or their mixture at different concentrations were obtained by diluted with distilled water ranging from 0.0001 to 0.1 mg/mL^−1^. After that, they were mixed with the pre-configured pyrene solution (6 × 10^−5^ M), and the concentration of pyrene in all the sample solutions was maintained as 6 × 10^−7^ M. Prior to measurement, the solutions were equilibrated at room temperature in the dark for 24 h. During the detailed operation process, different samples were tested with the fluorescence spectrometer, and the concentration and fluorescence intensity were recorded. To enhance the accuracy, each experimental point was repeated three times. In order to calculate the CMC values of the polymers and their mixture, the relationship between the solution concentration of polymers and fluorescence intensity was investigated.

### 2.6. Preparation and Characterization of the Blank and DOX-Loaded Mixed Micelles

The blank mixed micelles formed from PDEAEMA-PPEGMA and PCL-PPEGMA were prepared by the solvent evaporation method [17]. The mixed polymers (PDEAEMA-PPEGMA 15 mg, PCL-PPEGMA 15 mg) were dissolved into 10 mL of acetone and added to 30 mL of deionized water drop by drop under agitation; then, acetone was volatilized under stirring at 25 °C for 24 h. After being filtered by a 0.45 μm membrane filter, the solution of blank mixed micelles with a concentration of 1 mg/mL was obtained. DLS was used to determine the micellar sizes and zeta potentials of a series of blank mixed micellar solutions with pH ranging from 3 to 10 prepared by HCl or NaOH (0.1 M).

The DOX-loaded mixed micelles were fabricated by the method of diafiltration [26,38,39]. In brief, DOX (5–15 mg) in which the hydrochloride was removed by TEA (0.02 mL per 10 mg DOX·HCl) and mixed polymers (PDEAEMA-PPEGMA 15 mg, PCL-PPEGMA 15 mg) were dissolved into 10 mL of DMSO and then stirred for 12 h. The mixture was transferred to a dialysis bag (3500–4000 Da) and dialyzed against for 48 h with 1 L of deionized water at room temperature. After that, agglomerated particles in micelles were removed by filtration with a 0.45 μm membrane filter. During the dialysis process, the two amphiphilic block polymers were intertwined to form self-assembled micelles with PCL/PDEAEMA cores and PPEGMA shells.

The following formulas (1) and (2) were used to define and calculate the DOX LC and EE, respectively [39]. First, 1.0 mg of DOX-loaded micelles was added in 10 mL of DMSO; then, the solution was analyzed by a UV-vis spectrophotometer (UV9600, Shanghai, China) at 480 nm using a standard calibration curve experimentally gained by the DOX at different concentrations in DMSO solutions.

(1) $$\mathrm{LC}\left(\%\right)=\frac{\mathrm{Weight}\;\mathrm{of}\;\mathrm{loaded}\;\mathrm{drug}}{\mathrm{Weight}\;\mathrm{of}\;\mathrm{drug}\;\mathrm{loaded}\;\mathrm{micelle}}\times 100\%$$

(2) $$\mathrm{EE}\left(\%\right)=\frac{\mathrm{Weight}\;\mathrm{of}\;\mathrm{loaded}\;\mathrm{drug}}{\mathrm{Weight}\;\mathrm{of}\;\mathrm{drug}\;\mathrm{in}\;\mathrm{feed}}\times 100\%$$

### 2.7. In Vitro Release of DOX

The in vitro controlled release curves of the DOX-loaded mixed micelles self-assembled from PDEAEMA-PPEGMA and PCL-PPEGMA were measured in different phosphate-buffered saline (PBS, pH 5.0 and pH 7.4) [39]. A total of 5 mg of lyophilized DOX-loaded micelles were dissolved in 5 mL of solution and put into a 3500–4000 Da dialysis bag after stabilizing for 1 h. Then, the dialysis bag was immersed in 45 mL of PBS buffer solution at 37 °C (body temperature) and stirred at 100 rpm in a water bath. At specific moments, the solution (4 mL) was sampled to analyze the amount of the DOX, and 4 mL of fresh PBS was added at once. The samples were determined by the UV-vis spectrophotometer at 480 nm, and the following formula (3) was used to calculate the cumulative drug release percent (*E*_r_):

(3) $$E_{r}\left(\%\right)=\frac{V_{e}\sum_{1}^{n-1}C_{i}+V_{0}C_{n}}{m_{\mathrm{drug}}}\times 100\%$$

where *V*_e_ = 4 mL; *V*_0_ = 50 mL; *C*_n_ is the DOX concentration of the n_th_ sample; and *m*_drug_ is the DOX amount in the micelles.

### 2.8. Cytotoxicity Test

The cytotoxic influences of the polymer PDEAEMA-PPEGMA, DOX-loaded mixed micelles, and free DOX were evaluated against HepG2 cells by MTT analysis [40]. Firstly, the HepG2 cells were inoculated into a 96-well plate at a density of 1.0 × 10^4^ cells per well and incubated for 24 h under 5% CO_2_ atmosphere at 37 °C. Secondly, the micelles with a range of desired concentrations were added into the wells for 48 h, and the fresh medium was utilized as the control group. The cells were incubated for another 4 h after the addition of MTT solution (20 μL, 5 mg/mL). Then, the purple formazan crystals were dissolved in 200 μL of DMSO, which was then shaken for 15 min at 150 rpm. In the end, the absorbance at 490 nm was measured by a microplate reader (SpectraMax M5 Microplate reader, Molecular Devices, USA). The following formula (4) was used to calculate the cell viability (%):

(4) $$\mathrm{Cell}\;\mathrm{viability}=\frac{A_{\mathrm{sample}}-A_{\mathrm{blank}}}{A_{\mathrm{control}}-A_{\mathrm{blank}}}\times 100\%$$

where *A*_control_ and *A*_sample_ represent the absorbance of the wells treated with different samples, and *A*_blank_ represents the absorbance of wells with neither cells nor samples.

### 2.9. DPD Simulations

The formation of the DOX-loaded mixed micelles, their microstructures and detailed DOX distribution, and the pH-responsive dynamics process of DOX were simulated by coarse-grained simulation with the dissipative particle dynamics (DPD) approach, which had been explored in other anticancer drug delivery systems [39,41]. The polymers PDEAEMA-PPEGMA and PCL-PPEGMA were separated into four types of beads: PEG (purple), MAA (pink), DEA (blue), and PCL (yellow). The DOX (Color: green) was separated into three DOX1 beads, one DOX2 bead, and one DOX3 bead. A bead of WATER (mint) consisted of six water molecules. In the acidic environment, DEAH and DOX3H were used to represent the ionized pH-sensitive DEA and DOX3 beads; correspondingly, the ionization of PDEAEMA-PPEGMA was PDEAEMAH-PPEGMA, and the ionization of DOX was DOXH (Figure S1).

According to our previous method, the interaction parameters (*a_ij_*), listed in Table S1, were calculated by Discovery, Amorphous, and Forcite Cell at 298.15 K in Materials Studio 7.0 (Accelrys Inc., San Diego, CA, USA. A 30 × 30 × 30 *r*_c_^3^ cubic simulation box with a periodic boundary condition was utilized. The radius of each bead was 3.7 Å, and the volume and average mass of the beads were about 210 Å^3^ and 123 amu. When the density of the beads was 3, the cut-off semi diameter *r*_c_ between two beads was 8.6 Å. In our simulations, the integral time step was 0.05 ns, and the spring constant was installed to 4.0. The DPD module in the commercial software package (Materials Studio 7.0, Accelrys Inc., San Diego, CA, USA) was executed in all simulations.

### 2.10. Statistical Analysis

We carried out statistical analysis using a two-sample Student’s *t*-test with unequal variance. *p* < 0.05 was considered statistically significant.

## 3. Results

### 3.1. Synthesis and Characterization of the PDEAEMA-PPEGMA and PCL-PPEGMA Polymers

**Scheme 2:**
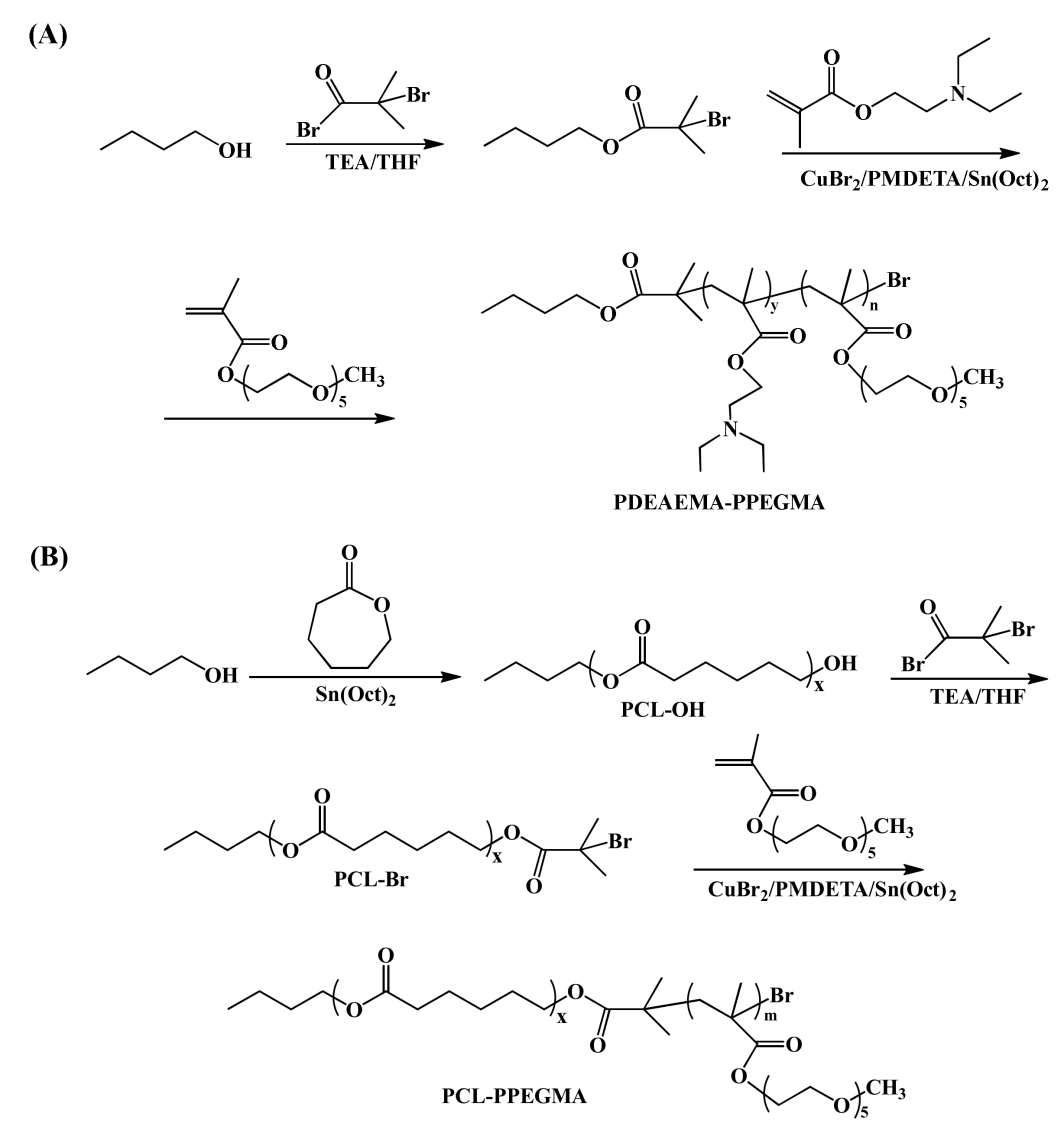
Synthetic routes for poly (*N,N*-diethylaminoethyl methacrylate)-*b*-poly(poly(ethylene glycol) methyl ether methacrylate) (PDEAEMA-PPEGMA) (**A**) and polycaprolactone-*b*-poly (poly(ethylene glycol) methyl ether methacrylate) (PCL-PPEGMA) (**B**).

Through the ARGET ATRP of DEAEMA and PEGMA segments, the pH-sensitive amphiphilic polymer PDEAEMA-PPEGMA was well synthesized (Scheme 2A). The small molecule initiator n-butanol 2-bromoisobutyrate was prepared by n-butanol with 2-bromoisobutyryl bromide using TEA as the acid binding agent, and then PDEAEMA-PPEGMA was synthesized using n-butanol 2-bromoisobutyrate as the initiator. Scheme 2B showed another amphiphilic polymer, PCL-PPEGMA, which was obtained by the ROP of ε-CL followed by the ARGET ATRP of PEGMA. Using n-butanol as the initiator, PCL-OH was synthesized by the ROP of ε-CL, which was brominated to become the macroinitiator PCL-Br. Finally, PCL-PPEGMA was polymerized by ARGET ATRP. By changing the mole ratio of PEGMA, three kinds of PCL-PPEGMA were synthesized, leading to different hydrophilic block lengths in the micelles.

**Figure 1:**
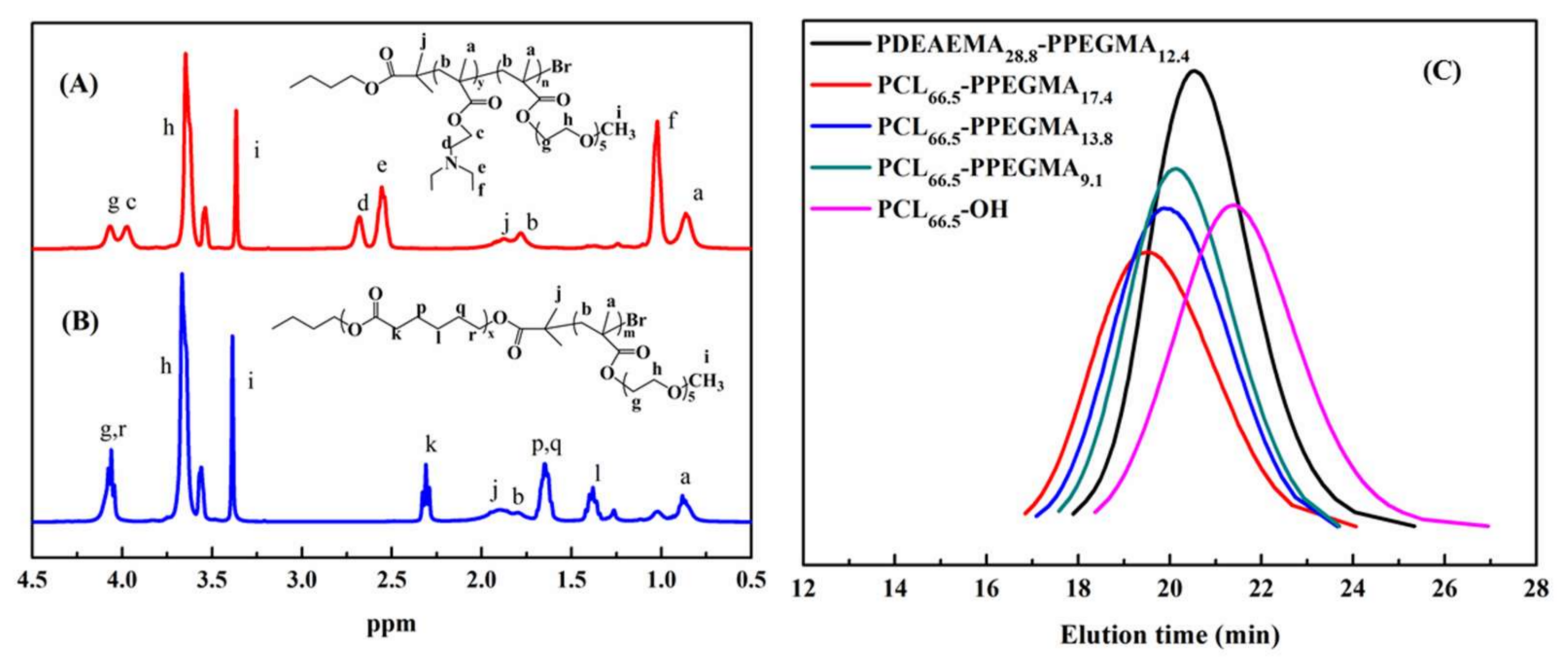
^1^H NMR spectra of PDEAEMA-PPEGMA (**A**) and PCL-PPEGMA (**B**) in CDCl_3_; gel permeation chromatographic (GPC) traces (**C**) of PDEAEMA-PPEGMA, PCL-PPEGMA, and PCL-OH.

The chemical structures of PDEAEMA-PPEGMA and PCL-PPEGMA were characterized by ^1^H NMR, which are shown in Figure 1A,B. The signals at 1.20 (f), 2.55 (e), and 2.65 (d) ppm were ascribed to -CCH_3_, CH_3_CH_2_NCH_2_-, and CH_3_CH_2_NCH_2_- on the side chain of the PDEAEMA unit, respectively. The signals at 4.3 (g) ppm, 3.67 (h) ppm, and 3.42 (i) ppm were ascribed to -COO-CH_2_-, -CH_2_CH_2_O-, and CH_3_O- of PPEGMA, respectively. The peak at 1.85 ppm (j) belonged to methane hydrogen of -CH_2_C(CH_3_)_2_Br in PDEAEMA and PPEGMA (Figure 1A). The signals at 2.35 (k) ppm, 1.60 (p, q) ppm, 1.40 (l) ppm, and 4.16 (r) ppm were the peaks of PCL (Figure 1B). The ^1^H NMR spectra of intermediate products n-butanol 2-bromoisobutyrate, PCL-OH, and PCL-Br are shown in Figures S2–S4.

The average molecular weights (*M*_n_) of these polymers were determined by GPC as well as estimated by ^1^H NMR spectra, and the results are displayed in Table 1. According to the integration ratio values of the peaks of PCL, PDEAEMA, and PPEGMA, the degrees of polymerization of PCL-OH, PDEAEMA, PPEGMA, and their average molecular weight (*M*_n, NMR_) were calculated. According to the feed ratio of monomers to initiator, the measured *M*_n, NMR_ and *M*_n_, _GPC_ values were close to the theoretical values *M*_n_ and _TH_, and the molecular weight distribution of the polymers was narrow, as the *M*_w_/*M*_n_ were all less than 1.3. The GPC traces of PDEAEMA-PPEGMA, PCL-PPEGMA, and PCL-OH in Figure 1(C) exhibited narrow unimodal distribution, suggesting that the ARGET ATRP and ROP processes were well-controlled.

### 3.2. CMC Values

**Figure 2:**
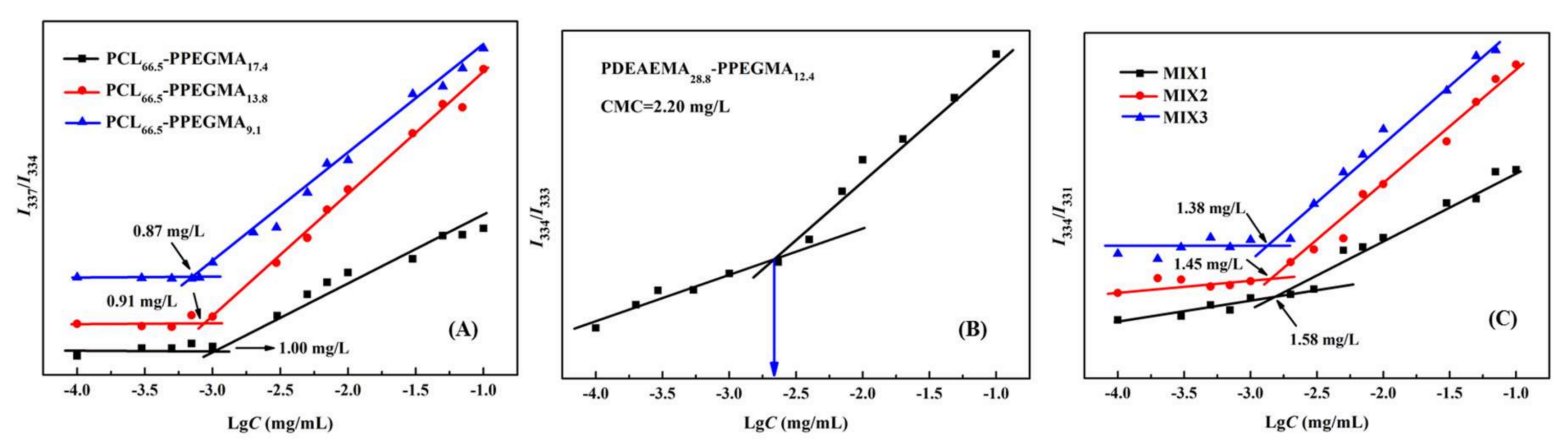
Different ratios of pyrene in neutral aqueous solution for PCL-PPEGMA (**A**), PDEAEMA-PPEGMA (**B**), and mixed polymers (**C**) at various concentrations.

It is generally acknowledged that the CMC value is one of the most significant parameters of amphiphilic polymers. Micelles that have low CMC values would remain stable in the blood circulation system and have less drug leakage, thus decreasing the toxic side effects [9]. In this study, in order to obtain stable multifunctional micelles and to decrease the CMC values, the polymers of PDEAEMA-PPEGMA (PDEAEMA_28.8_-PPEGMA_12.4_) and PCL-PPEGMA (PCL_66.5_-PPEGMA_17.4_, PCL_66.5_-PPEGMA_13.8_, PCL_66.5_-PPEGMA_9.1_) and their mixed micelles MIX1, MIX2, and MIX3 were prepared; then, their CMC values were measured by fluorescence spectroscopy. For the blank mixed micelles, MIX1 was composed of PDEAEMA_28.8_-PPEGMA_12.4_ and PCL_66.5_-PPEGMA_17.4_, MIX2 was composed of PDEAEMA_28.8_-PPEGMA_12.4_ and PCL_66.5_-PPEGMA_13.8_, MIX3 was composed of PDEAEMA_28.8_-PPEGMA_12.4_ and PCL_66.5_-PPEGMA_9.1_, and all the mass ratios of PCL-PPEGMA and PDEAEMA-PPEGMA were set at 1:1. Figure 2 showed the *I*_337_/*I*_334,_ *I*_334_/*I*_333_ and *I*_334_/*I*_331_ ratios of pyrene in different concentrations of the polymers in neutral aqueous solution. The results found that in neutral aqueous solution, the CMC values of polymers PCL_66.5_-PPEGMA_17.4_, PCL_66.5_-PPEGMA_13.8_, and PCL_66.5_-PPEGMA_9.1_ were 1.00 mg/L, 0.91 mg/L, and 0.87 mg/L, respectively (Figure 2A); PDEAEMA_28.8_-PPEGMA_12.4_ was 2.20 mg/L (Figure 2B), and MIX1, MIX2, and MIX3 were 1.58 mg/L, 1.45 mg/L and 1.38 mg/L, respectively (Figure 2C). Our previous work showed that the CMC values of mixed micelles formed by MPEG-PDEAEMA and MPEG-PCL were determined as 12.59 mg/L, 6.31 mg/L, and 4.47 mg/L [26]. However, the CMC results of MIX1, MIX2, and MIX3 prepared in this study were far lower, suggesting that the mixed micelles formed by PDEAEMA-PPEGMA and PCL-PPEGMA might have superior stability under neutral conditions, which verified our conception that using a hydrophilic brush PPEGMA block with denser side chains instead of a linear MPEG block could effectively decrease the CMC values of the micelles, thus improving the stability and longevity of micelles during biological circulation.

### 3.3. Particle Sizes and Zeta Potentials of the Blank Mixed Micelles

**Figure 3:**
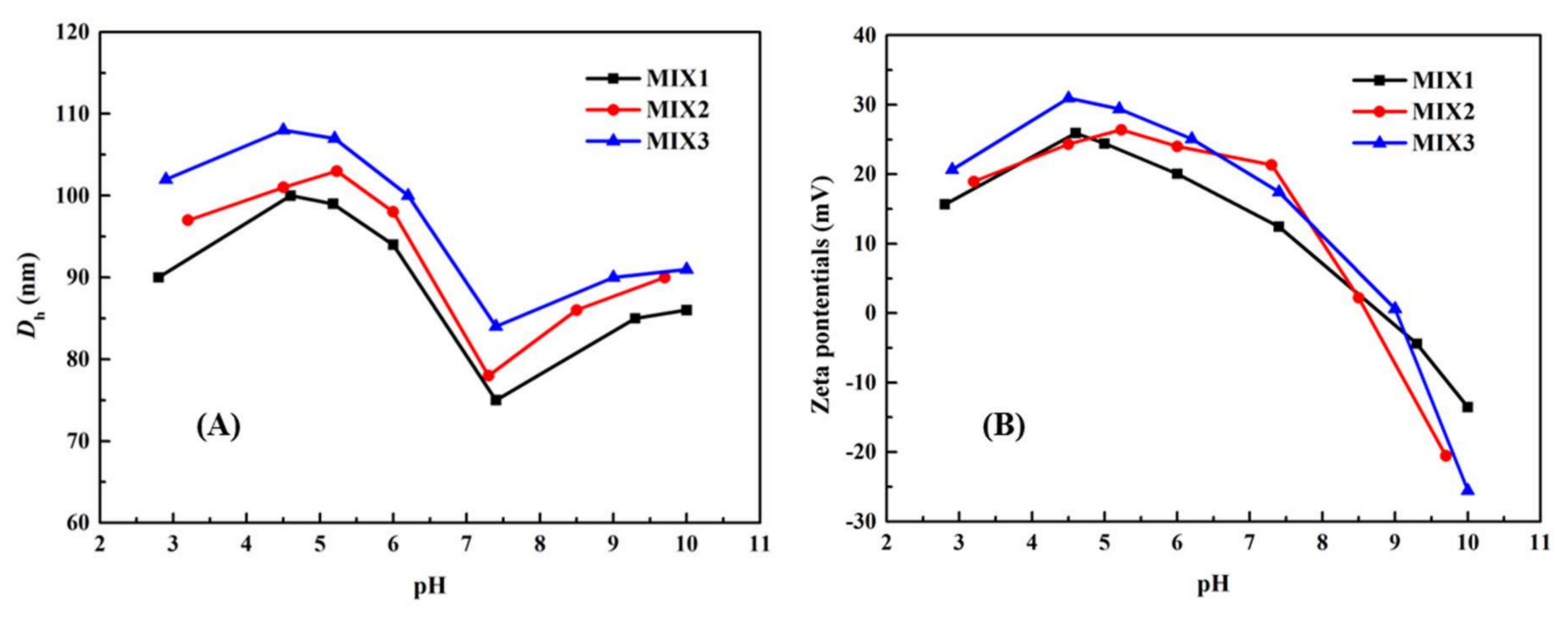
(**A**) Particle sizes and (**B**) zeta potentials of the polymeric mixed micelles dependence on pH values in aqueous solution.

The micellar sizes and zeta potentials of blank mixed micelles were measured at different pH ranging from 2 to 10 by DLS, and the results are shown in Figure 3. In Figure 3A, for all the three micelles, when pH > 7.4, the PDEAEMA block (p*K*_a_ ≈ 6.9) began to deprotonate slowly, resulting in the aggregation of the polymeric micelles, and some micelles might be transformed into larger micelles, leading to an increase in the sizes of the micelles. When the pH value reduced from 7.4 to 4.0, as the pendant tertiary amine groups in PDEAEMA block gradually were protonated and their solubility changed from hydrophobicity to hydrophilicity, augmentation of the effective particle sizes of micelles was obvious. When the pH value declined below 4, the particle sizes slowly decreased. This was because the PDEAEMA block was fully protonated, which led to the amphiphilic PDEAEMA-PPEGMA completely changing into the hydrophilic one, resulting in the aggregation of the micelles; dissolving into the solution, and new little sizes of micelles with single amphiphilic PCL-PPEGMA polymer were formed. The particle sizes for the three micelles were all changed with pH values and showed a similar tendency. However, the ratios of PCL blocks in the same weight of amphiphilic polymers PCL_66.5_-PPEGMA_17.4_, PCL_66.5_-PPEGMA_13.8_, and PCL_66.5_-PPEGMA_9.1_ were different, which resulted in different micellar sizes of mixed micelles in aqueous solution.

Figure 3B displayed the changes of the zeta potentials values of the blank mixed micelles at different pH values. The zeta potential decreased sequentially from the positive charge to the negative one when the pH values increased from 7.4 to 10.0, which might cause the aggregation of the micelles, resulting in the increase of the particle sizes. When the pH values dropped from 7.4 to 4.0, the zeta potential increased because the PDEAEMA block continuously protonated and the particle sizes grew. When the pH values dropped from 4 to 2, the zeta potentials downregulated mildly due to the PDEAEMA-PPEGMA being gradually dissolved into the solution. Despite the PPEGMA chain lengths in PCL-PPEGMA being different, the zeta potentials of the three blank mixed micelles showed similar trends relying on the pH values. The reason might be due to the same proportion of PDEAEMA-PPEGMA in MIX1, MIX2, and MIX3.

In summary, both the micellar sizes and the zeta potentials of the three blank mixed micelles were changed relying on the pH values obviously, which showed that the prepared mixed micelles had good pH-responsive behavior.

### 3.4. Characterization of DOX-Loaded Mixed Micelles

Nowadays, computational simulations have become a powerful analytical approach for experimentally derived results. DPD simulations were carried out to investigate how the feed DOX concentrations affect the aggregate morphologies of the DOX-loaded mixed micelles. The formation process of the DOX-loaded mixed micelles, MIX1, was shown in Figure S5. We observed that the components of the system gradually aggregated and finally formed into large spherical micelles at equilibrium states.

DLS was used to detect the polydispersity index (PDI) and particle sizes; UV-vis spectrophotometry was used to detect the LC and EE of the DOX-loaded mixed micelles (Table 2). For all the DOX-loaded mixed micelles, the PDIs were all less than 0.3, indicating that these micelles had good physical performance and narrow unimodal distribution. The DOX-loaded micelles formed by MIX1 at different feed ratios were investigated. It was found that the weight feed ratio of DOX/mixed polymers had an important impact on the particle sizes and LC of the DOX-loaded mixed micelles. With more DOX fed to the micelles, larger particle sizes and higher LC would be obtained. Therefore, in order to get better LC, 15 mg/30 mg DOX/mixed polymers were adopted to study the properties of the different mixed micelles. The LC and EE of micelles formed by MIX1 were 31.23% and 90.82% respectively, which were much bigger than those of MIX2 (26.53% and 72.22%) and MIX3 (23.36% and 60.96%).

**Figure 4:**
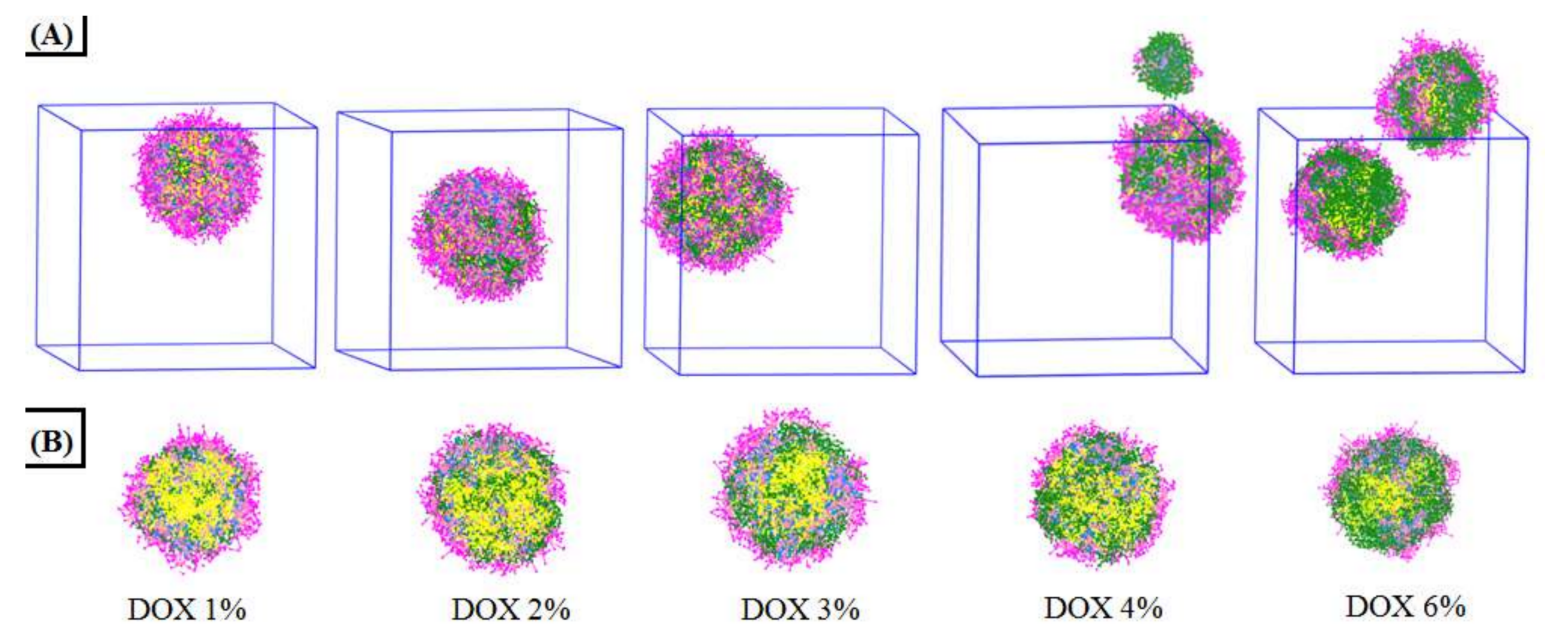
Equilibrium states (**A**) and cross-setion views (**B**) of the DOX-loaded mixed micelles at different DOX concentrations.

Maintaining the mixed polymeric and the same volume fraction, with the volume fraction of DOX in the range of 1% to 6%, the aggregation morphologies and the cross-section views of the DOX-loaded mixed micelles at equilibrium states are shown in Figure 4. It was found that the DOX concentrations do influence the aggregate morphologies and DOX distribution of the mixed micelles. When the volume fraction of DOX was at 1%, a single micellar sphere with most of the DOX molecules was encapsulated in the inner core, and a little of DOX was exposed at the surface. The retention in the micellar inner core increased with the increase of DOX concentration until it reached saturation (DOX 3%). When the DOX concentration increased to 4% and 6%, in which the drug-loaded capacity of the micellar sphere had exceeded the saturation, the mixture formed into two smaller micelles. At the same time, more and more DOX was exposed on the surface of the micellar spheres. The cross-section views shown in Figure 4B further demonstrated the saturation process of DOX in the micellar inner core when the DOX concentration increased from 1% to 6%.

**Figure 5:**
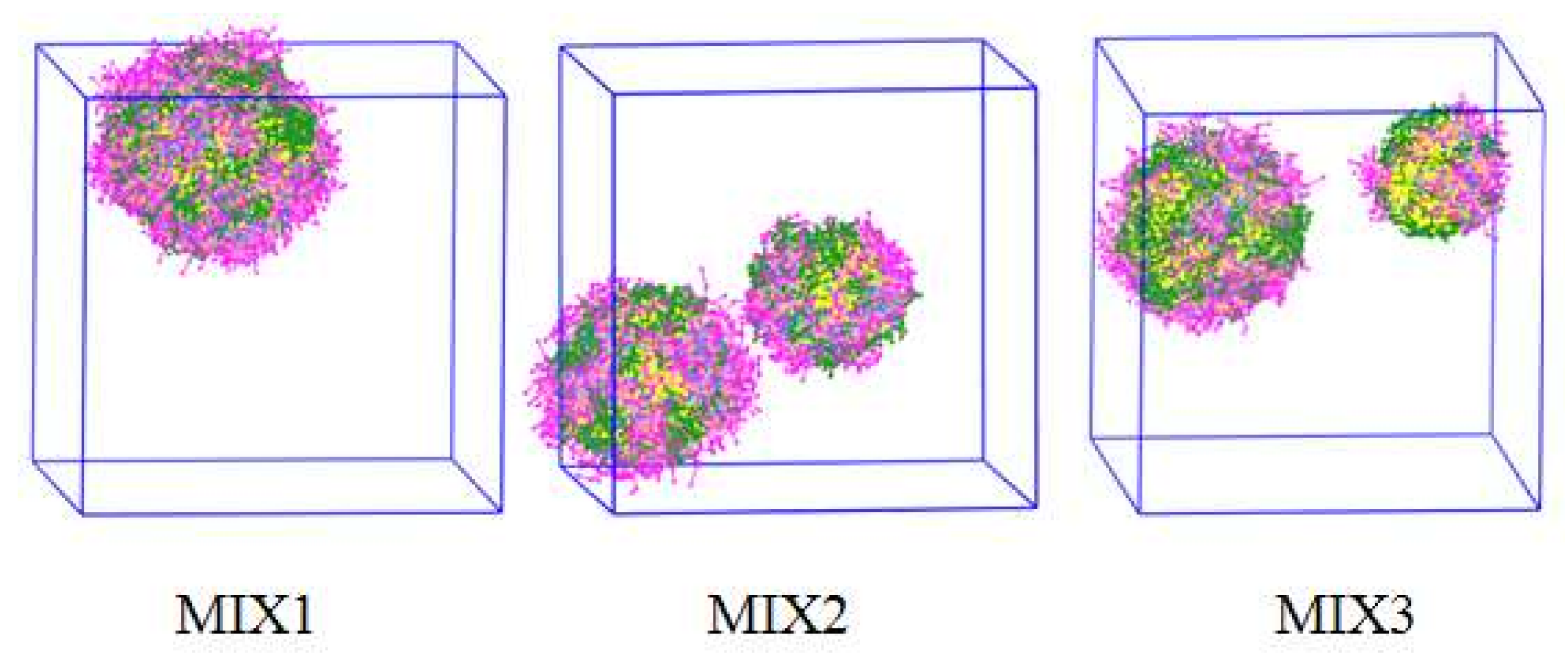
Morphologies of MIX1, MIX2, and MIX3 at 120,000 steps with 3% volume fraction of PDEAEMA-PPEGMA, 3% volume fraction of PCL-PPEGMA, and 3% volume fraction of DOX.

MIX1, MIX2, and MIX3, three kinds of DOX-loaded micelles, had hydrodynamic diameters (*D*_h_) of 253, 218, and 202 nm (Table 2), respectively. The DPD simulation results of the DOX-loaded mixed micelles of MIX1, MIX2, and MIX3 are presented in Figure 5. It was observed that a large single micelle was formed in the MIX1 system, while two smaller micelles were formed in the systems of MIX2 and MIX3, which were in accordance with the actual experimental results summarized in Table 2, in which the particle sizes of MIX1 were larger than those of MIX2 and MIX3.

**Figure 6:**
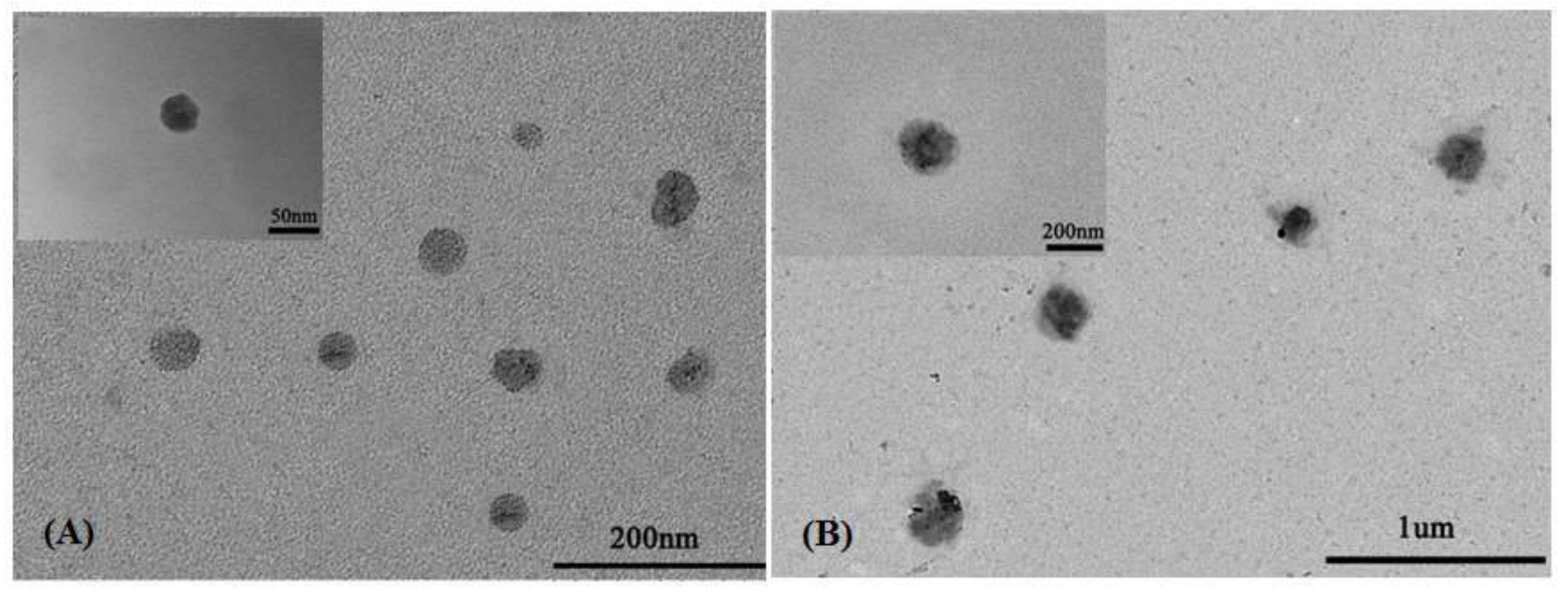
TEM images of blank MIX1 micelles (**A**) and DOX-loaded MIX1 micelles (**B**).

To further confirm the mixed micellar sizes of the mixed micelles, the TEM images of blank mixed micelles MIX1 (the feed ratio of DOX/mixed polymers 0/30) and DOX-loaded mixed micelles MIX1 (the feed ratio of DOX/mixed polymers 15/30) are presented in Figure 6. It could be observed that both the blank mixed micelles and the DOX-loaded mixed micelles showed spherical morphology, and the particle sizes estimated from TEM for the blank mixed micelles and DOX-loaded mixed micelles were about 50 nm and 200 nm, respectively, which were in quite good agreement with those estimated from DLS, and the slight difference was due to the different measured principles and preparing methods of test samples between DLS and TEM.

In our previous work, the mixed micelles formed by MPEG-PCL and MPEG-PDEAEMA had the biggest LC and EE achieved at 26.79% and 63.19% [26]. The study herein seemed to reach the target to obtain well potential DOX-loaded mixed micelles with lower CMC values, good stability, as well as high LC and EE performance by using PDEAEMA-PPEGMA and PCL-PPEGMA mixture as the carriers.

### 3.5. DOX Release Performance of DOX-Loaded Mixed Micelles

In this study, as the polymeric block PDEAEMA could be the protons acceptor and turn their conformation state from hydrophobic to hydrophilic, the designed DOX-loaded mixed micelles were sensitive to pH, and DOX would be released in acidic aqueous solution.

**Figure 7:**
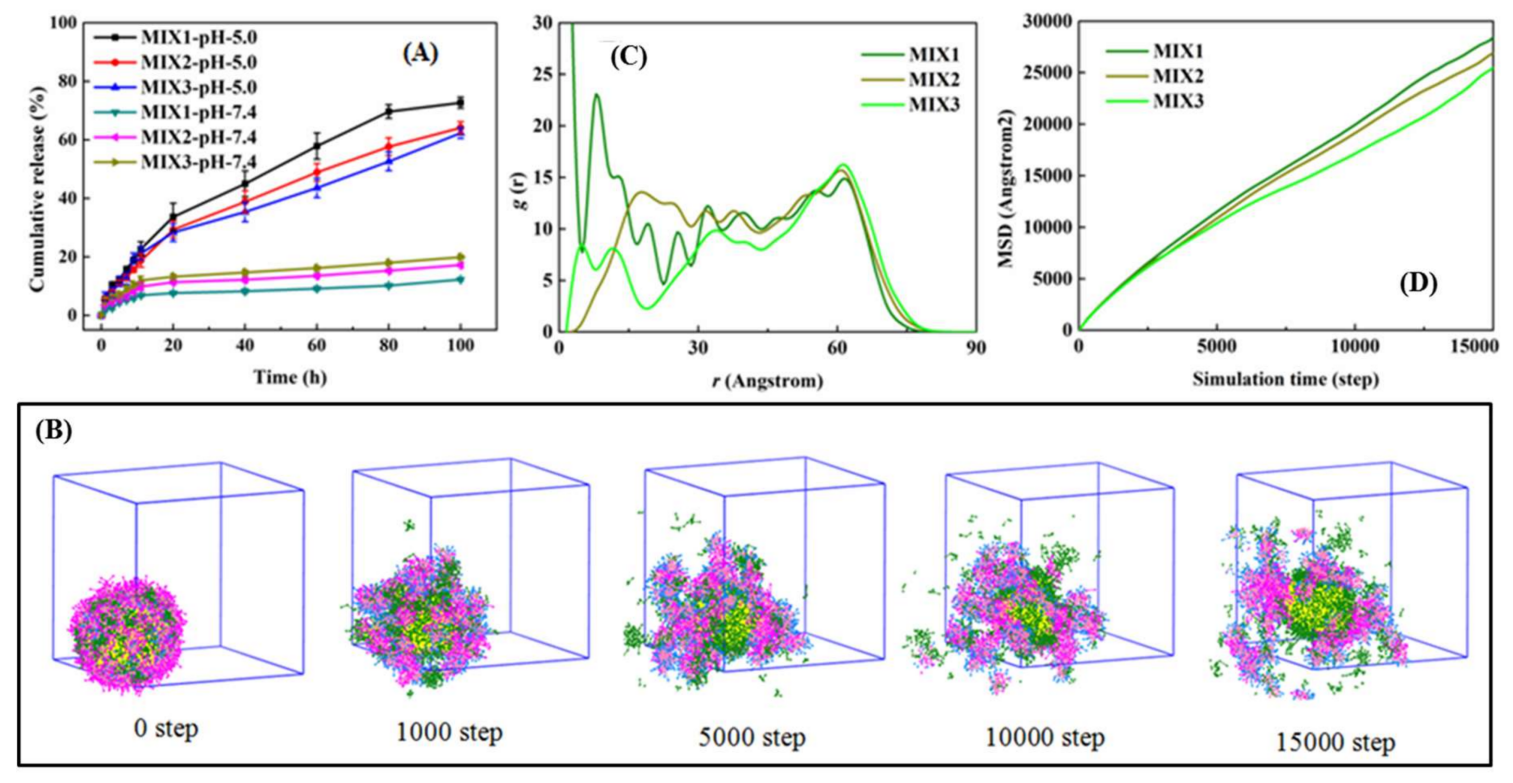
In vitro drug release profiles (**A**) at pH 7.4 and pH 5.0; (**B**) DOX release process of MIX1 (LC, 31.23%) in acidic aqueous solution; (**C**) radial distribution functions (RDF) curves between DOX and the micellar center; (**D**) mean square displacements (MSD) curves of the DOX-loaded mixed micelles MIX1 (LC, 31.23%), MIX2 (LC, 26.53%), and MIX3 (LC, 23.36%).

The proportion of the hydrophilic block of PEGMA presented an immense effect on the expected in vitro drug release of the designed micelles. The in vitro DOX release behavior of the DOX-loaded mixed micelles (the feed ratio of DOX/mixed polymers 15/30) formed by MIX1, MIX2, and MIX3 were studied under at 37 °C at pH 5.0 and pH 7.4, as shown in Figure 7A. As expected, when the pH values dropped from 7.4 to 5.0, the received DOX-loaded mixed micelles displayed strong pH-responsive behavior, and it was obvious that the lower pH had a faster release rate.

Regarding MIX1, the micelles only released approximately 5% of DOX at pH 7.4 in 6 h; after that, the release rate increased tardily and tended toward stability, to around 7% of DOX released in 20 h, and less than 13% after 100 h, suggesting that the micelles could remain stable for a long time at pH 7.4, and the release of DOX from micelles was low in the blood circulation due to the good protection of the drug. It meant that the micelles had good stability and less leakage of DOX in neutral conditions. The release rates of the micelles, which were formed by MIX2 and MIX3, exhibited slightly faster than that of MIX1 at pH 7.4, due to the lower percentage of PEGMA block in MIX2 and MIX3, in which more DOX was exposed outside the surfaces of the DOX-loaded mixed micelles. This conclusion could also be supported by the microstructures of the DOX-loaded mixed micelles and their DOX distribution (Figure S6). It could be seen that although the LC of MIX1 was the largest, a majority of the DOX was encapsulated into the inner core instead of at the interface between the inner core and PPEGMA shell, resulting in less DOX release at pH 7.4. As shown in Figure 7C, the radial distribution functions (RDF) could also be used to quantify the distribution of DOX. At the surfaces of the micelles, the distribution probability (*g*(*r*)) of DOX in MIX1 was slightly less than that of MIX2 and MIX3, indicating the relatively slower release of DOX at pH 7.4.

Conversely, the drug release rate of DOX was expedited at pH 5.0, which was obviously because of the swelling of the micelles, on account of the tertiary amine groups of PDEAEMA protonated. The cumulative release of the micelles formed by MIX1 was about 10% at 3 h, 20% in 10 h, nearly 45% in 40 h, and approximately 75% in 100 h, demonstrating that the DOX-loaded micelles formed by MIX1 could effectively reduce the initial burst and achieve slow and long-term drug release. Under the weak acid condition, the DOX release rates of the micelles, which were formed by MIX2 and MIX3, were both slightly slower than that of MIX1, because of the decrease of the pH sensitivity of the micelles, and the shorter hydrophilic PEGMA block of MIX2 and MIX3 increased the proportion of non pH-responsive hydrophobic PCL block, which could prevent the fast release of the drug.

The release process of DOX in the acidic aqueous solution could be investigated at the mesoscopic scale by DPD simulations. For example, the dynamics release process of DOX for MIX1 (LC, 31.23%) was shown in Figure 7B. Initially, the DOX-loaded mixed micelles appeared as a consummate sphere, and the drug DOX had a good aggregation in the spherical core. As the solutions changed from neutral to acidic, the pH-responsive block PDEAEMA accepted protons and became ionized, causing the micelles to swell, and then DOX gradually released from the micelles. As the MSD value was defined as the distance that the beads moved to the second moment of their distribution from their original position within the specified time range, the diffusion behavior of DOX during the release process was analyzed by using the MSD values of DOX in our systems. From Figure 7D, it was seen at the beginning that for a simulation step less than 3000, the MSD values of MIX1, MIX2, and MIX3 showed little difference, but as the stimulation step increased, the MSD values of MIX1 gradually grew higher than those of MIX2 and MIX3, which coincided with the experimental results of the in vitro drug release tests at pH 5.0, as shown in Figure 7A.

Overall, by combining experiments with DPD simulations, it was found that most of the properties of the mixed micelles formed by MIX1 displayed superior drug release performance to those of MIX2 and MIX3, owing to the longer hydrophilic PEGMA block chains in PCL-PPEGMA, which provided more hydrophilic shell to improve the stability of the DOX-loaded mixed micelles in the normal environment and less hydrophobic PCL block to trigger the fast rate of drug release in the acidic environment.

To sum up, the in vitro drug release profiles and the DPD simulation results revealed that the DOX-loaded mixed micelles formed by PDEAEMA-PPEGMA and PCL-PPEGMA showed good pH-responsive behavior, providing less leakage and good stability in normal tissues (pH 7.4) and accelerating the drug-release behavior in the absence of an initial burst at tumor cells (pH 5.0). Therefore, as potential carriers, they could be used to deliver kinds of hydrophobic drugs with well-controlled release performance. From the performance results of mixed micelles, with the increase of hydrophilic blocks, the efficiency of drug loading was higher, and the release performance in vitro was preferable.

### 3.6. Cytotoxicity Test

**Figure 8:**
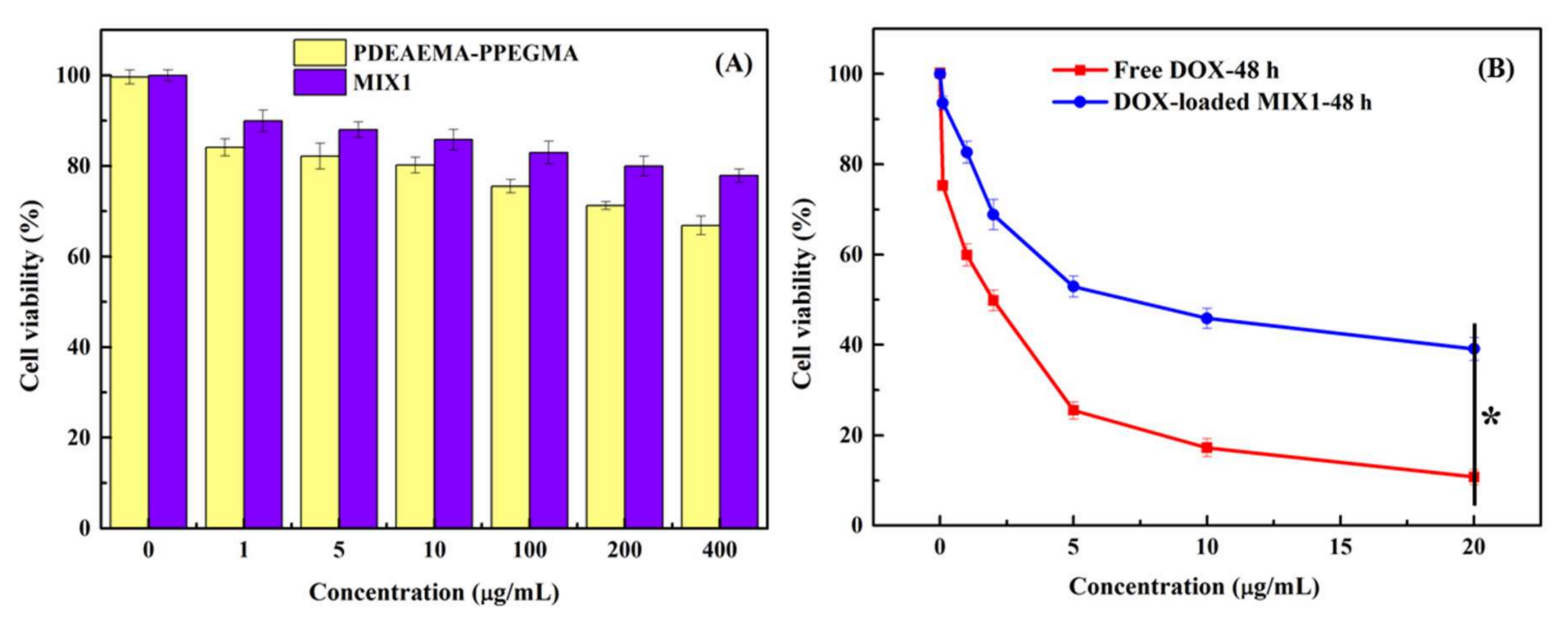
In vitro cytotoxicity of blank MIX1 and polymer PDEAEMA_28.8_-PPEGMA_12.4_ (**A**), free DOX and DOX-loaded mixed micelles (**B**) and in HepG2 cells. *p < 0.05.

The in vitro cytotoxicity tests of blank micelles, DOX-loaded mixed micelles, and free DOX in HepG2 cells were carried out by the MTT method. Figure 8A shows the cytotoxic results of blank mixed micelles MIX1 and polymer PDEAEMA_28.8_-PPEGMA_12.4_ in vitro cultivated with HepG2 cells for 48 h. As the concentration of MIX1 increased to 400 μg/mL, there was still over 80% cell survival, indicating that hypotoxicity was found for the blank micelles of MIX1 against HepG2 cells. The cytotoxicity of the polymer PDEAEMA_28.8_-PPEGMA_12.4_ was found to be a little higher than that of MIX1, and the cell viability was around 70% at the concentration of 400 μg/mL. It might be due to the existence of amine groups in the PDEAEMA unit, as this kind of polymer usually showed high electropositivity and resulted in the increase of toxicity. However, when MIX1 was used in the dilution of the PCL-PPEGMA, the whole mixed micelles showed no obvious cytotoxicity with over 80% cell survival, which met the requirements of the drug delivery carriers.

Figure 8B showed the in vitro cytotoxic results of DOX-loaded mixed micelles and free DOX cultivated with HepG2 cells for 48 h. The IC_50_ values of DOX-loaded mixed micelles and free DOX were 7.5 and 2.5 μg/mL, respectively. Compared with the free DOX, the in vitro cytotoxic effects of the DOX-loaded micelles MIX1 (the feed ratio of DOX/mixed polymers 15/30) were lower at the concentration of 20 μg/mL, because the cumulative DOX released from the DOX-loaded mixed micelles in 48 h was less than 20% at neutral pH. Therefore, compared with the free DOX, the cell-killing rate of DOX-loaded micelles was more moderate and slower, which was consistent with drug release profiles in vitro, preventing the DOX initial burst in the therapeutic process.

## 4. Conclusions

The PDEAEMA-PPEGMA and PCL-PPEGMA polymers were synthesized and confirmed by ^1^H NMR and GPC, and their self-assembled mixed micelles were applied for the delivery of anticancer drug. The CMC values of the mixed micelles achieved were 1.58 mg/L (MIX1), 1.45 mg/L (MIX2), and 1.38 mg/L (MIX3), respectively. The micellar sizes and zeta potentials of the blank mixed micelles were all dependent on the pH values obviously, indicating that mixed micelles formed from the mixture of PDEAEMA-PPEGMA and PCL-PPEGMA had good pH-responsive behavior. The LC and EE of the mixed micelles were 31.23% and 90.82% (MIX1), 26.53% and 72.22% (MIX2), and 23.36% and 60.96% (MIX3), indicating the high DOX-loading capacity of these mixed micelles. DPD simulations showed that the feed DOX concentrations affected the aggregation morphologies of the DOX-loaded mixed micelles, which could well explain the particle sizes and the DOX distribution. Both in vitro DOX release profiles and DPD simulation of these mixed micelles demonstrated their good pH-responsive behavior, which provided good stability and less leakage at pH 7.4 and accelerated drug release behavior at pH 5.0. The in vitro cytotoxicity tests showed that the polymer PDEAEMA-PPEGMA and the blank mixed micelles had good biocompatibility, and the cytotoxicity of DOX-loaded mixed micelles was consistent with the results of in vitro DOX release within 48 h. Therefore, the drug-loaded mixed micelles self-assembled from the two polymers PDEAEMA-PPEGMA and PCL-PPEGMA showed great potential for the application of delivering anticancer drugs with well-controlled release.

## Figures and Tables

**Table 1:** GPC and ^1^H NMR results of polymeric products.

Samples	*M*_n, NMR_^a^	*M*_n,GPC_^b^	*M*_n,TH_^c^	*M*_w_/*M*_n_^b^
PCL_66.5_-OH	6724	7120	6074	1.21
PDEAEMA_28.8_-PPEGMA_12.4_	9259	9820	10,261	1.08
PCL_66.5_-PPEGMA_9.1_	9603	10,460	9873	1.11
PCL_66.5_-PPEGMA_13.8_	11,013	11,619	11,373	1.13
PCL_66.5_-PPEGMA_17.4_	12,093	12,634	12,873	1.17

^a^ Calculated by ^1^H NMR spectra; ^b^ Measured by GPC in tetrahydrofuran (THF); ^c^ Calculated by theory analysis from the feed ratio of monomers to initiator.

**Table 2:** Size, polydispersity index (PDI), loading content (LC), and encapsulation efficiency (EE) of the DOX-loaded mixed micelles.

Sample	DOX/Polymer (mg/mg)	Size (nm)	PDI	LC (%)	EE (%)
MIX1	0/30	75	0.219	-	-
MIX1	5/30	170	0.208	12.46%	85.40%
MIX1	10/30	194	0.284	17.89%	65.36%
MIX1	15/30	253	0.293	31.23%	90.82%
MIX2	15/30	218	0.219	26.53%	72.22%
MIX3	15/30	202	0.255	23.36%	60.96%

## Supplementary Materials

The following are available online at https://www.mdpi.com/1999-4923/12/2/170/s1, Figure S1: Coarse grain models of PDEAEMA-PPEGMA, PCL-PPEGMA, DOX, water, ionized PDEAEMAH-PPEGMA and DOXH, Figure S2: 1H NMR spectrum of n-butanol 2-bromoisobutyrate, Figure S3: 1H NMR spectrum of PCL-OH, Figure S4: 1H NMR spectrum of PCL-Br, Figure S5: Morphologies of the mixed micelles at different simulation time with 3% volume fraction of PDEAEMA-PPEGMA, 3% volume fraction of PCL-PPEGMA, and 3% volume fraction of DOX, Figure S6: Equilibrium states (A) and cross-setion views (B) and RDF curves between different beads and the micellar center of the DOX-loaded micelles, Table S1: Interaction parameters aij between different beads used in DPD simulation.
