# Supplementary material for: pH-Sensitive Mixed Micelles Assembled from PDEAEMA-PPEGMA and PCL-PPEGMA for Doxorubicin Delivery: Experimental and DPD Simulations Study
Source: Pharmaceutics. 2020 Feb 18;12(2):170. doi: 10.3390/pharmaceutics12020170 (PMC7076365; doi:10.3390/pharmaceutics12020170)
Supplement: Supplementary file 1 [file pharmaceutics-12-00170-s001.pdf]

pharmaceutics

Supplementary Materials: pH-Sensitive Mixed Micelles Assembled from PDEAEMA-PPEGMA and PCL-PPEGMA for Doxorubicin Delivery: Experimental and DPD Simulations Study

Chufen Yang, Wenyao Liu, Jiayu Xiao, Cong Yuan, Yaoxi, Chen, Jianwei Guo, Hangbo Yue, Dongyu Zhu, Wenjing Lin, Shengqiu Tang, Xiaoying Dong

Table S1. Interaction parameters aij between different beads used in DPD simulation.

a_{ij}	PCL	MAA	DOX1	DOX2	DOX3	DOX3H	PEG	DEA	DEAH	WATER
PCL	25.00									
MAA	27.01	25.00								
DOX1	25.42	25.63	25.00							
DOX2	25.03	26.69	25.26	25.00						
DOX3	25.1	26.29	25.09	25.05	25.00					
DOX3H	83.78	41.87	21.56	23.25	—	25.00				
PEG	34.41	27.39	31.14	34.22	33.61	21.46	25.00			
DEA	25.95	30.44	27.66	26.36	25.70	—	30.58	25.00		
DEAH	96.82	31.14	31.77	37.04	—	24.34	22.82	—	25.00	
WATER	117.12	68.9	40.37	31.36	130.78	8.57	26.10	135.82	10.35	25.00

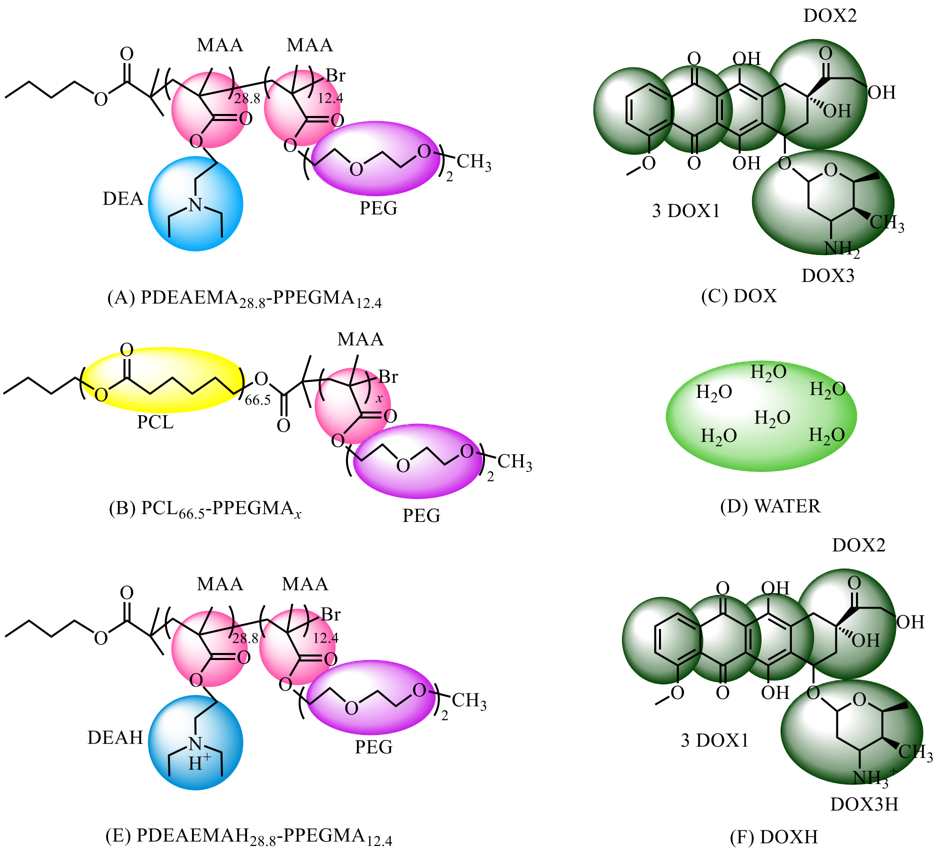

Figure S1. Coarse grain models of PDEAEMA-PPEGMA, PCL-PPEGMA, DOX, water, ionized PDEAEMAH-PPEGMA and DOXH.

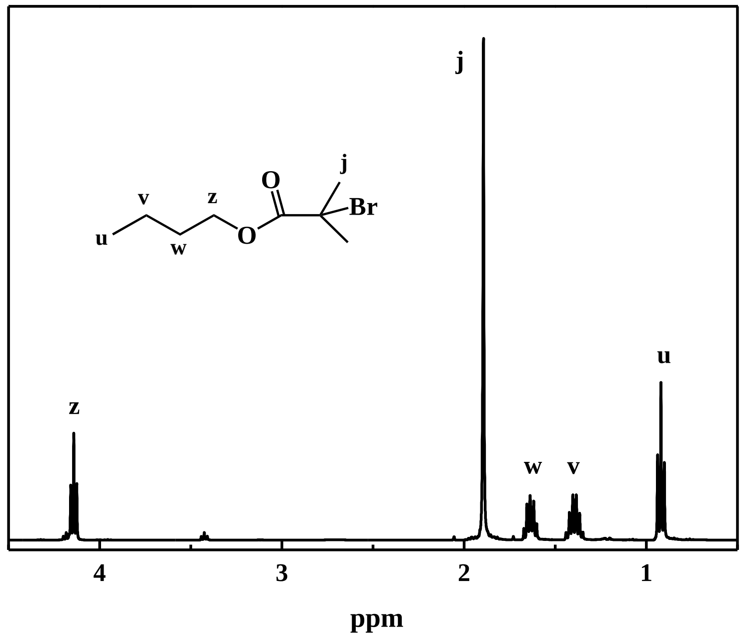

Figure S2. ^{1}H NMR spectrum of n-butanol 2-bromoisobutyrate.

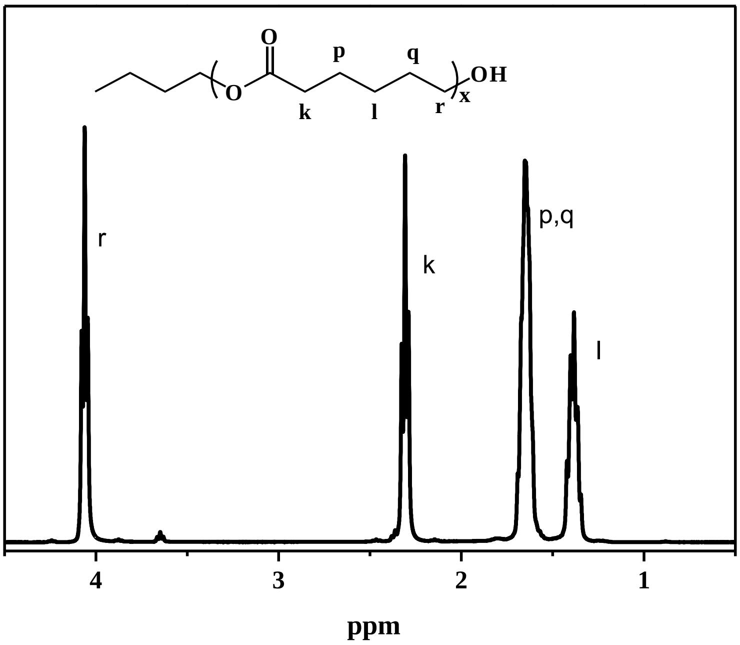

Figure S3. ^1H NMR spectrum of PCL-OH.

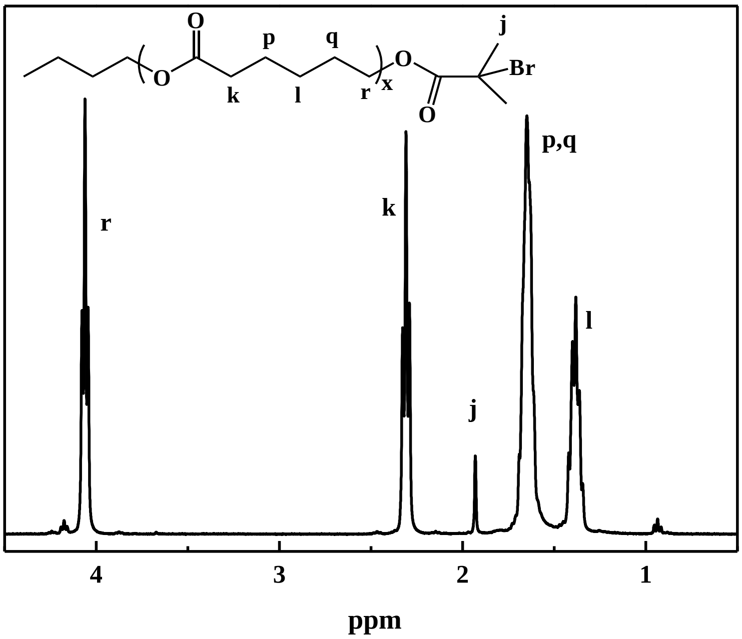

Figure S4. ^1H NMR spectrum of PCL-Br.

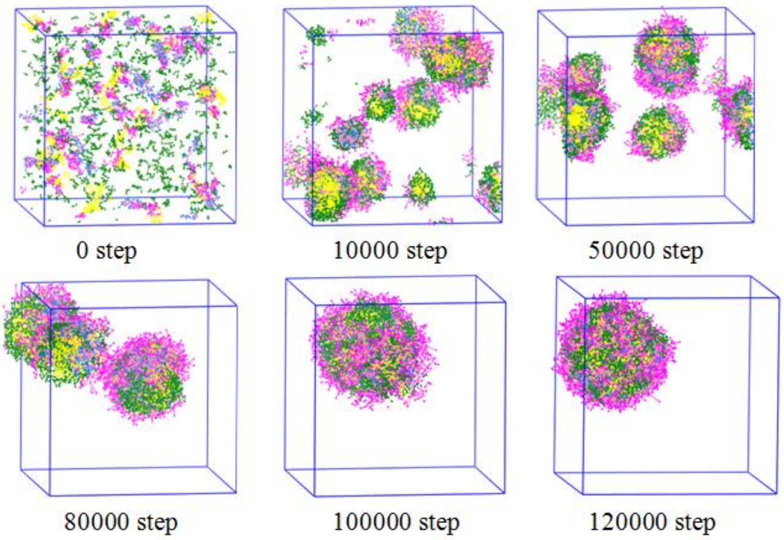

Figure S5. Morphologies of the mixed micelles at different simulation time with 3% volume fraction of PDEAEMA-PPEGMA, 3% volume fraction of PCL-PPEGMA, and 3% volume fraction of DOX.

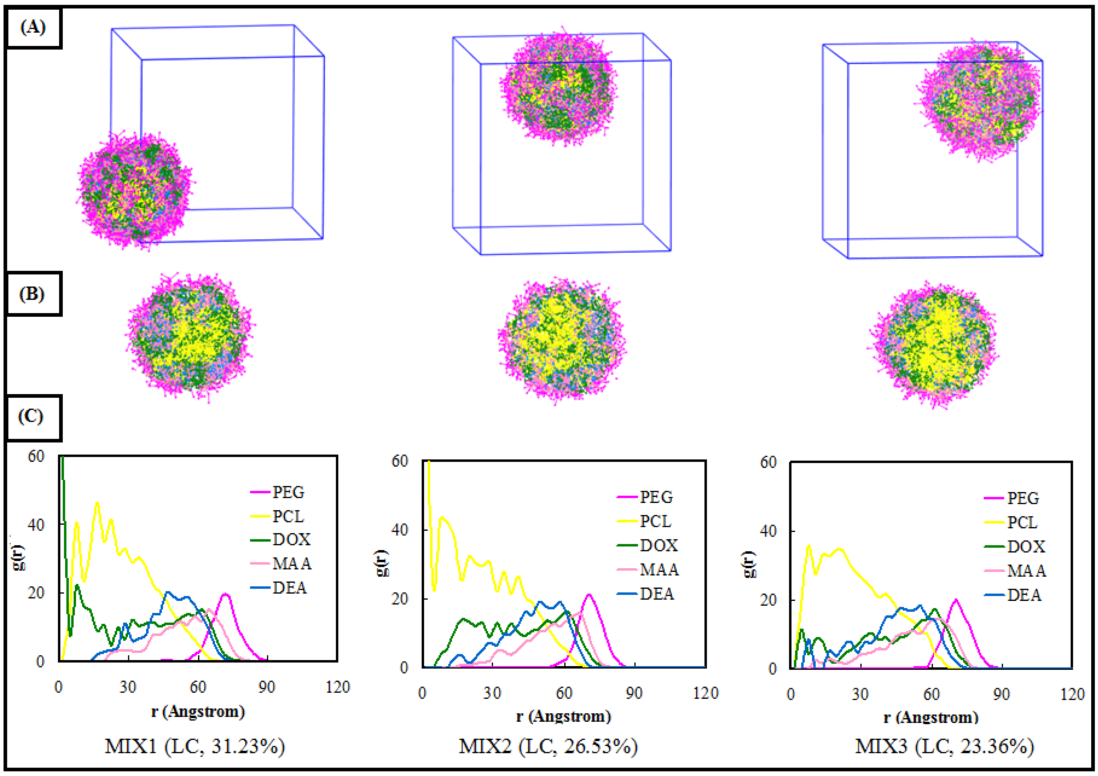

Figure S6. Equilibrium states (A) and cross-setion views (B) and RDF curves between different beads and the micellar center of the DOX-loaded micelles.
